# (*S*)-*N*-{1-[5-(4-Chloro­benzyl­sulfanyl)-1,3,4-oxadiazol-2-yl]eth­yl}-4-methyl­benzene­sulfonamide

**DOI:** 10.1107/S1600536811039134

**Published:** 2011-09-30

**Authors:** Tayyaba Syed, Shahid Hameed, Peter G. Jones

**Affiliations:** a Department of Chemistry, Quaid-i-Azam University, Islamabad 45320, Pakistan; b Institut for Anorganische und Analytische Chemie, Technische Universität Braunschweig, Hagenring 30, 38106 Braunschweig, Germany

## Abstract

The title compound, C_18_H_18_ClN_3_O_3_S_2_, adopts by folding the form of a distorted disc. Inter­planar angles are 29.51 (7) and 63.43 (7)° from the five-membered ring to the aromatic systems and 34.80 (6)° between these two latter rings. The absolute configuration was confirmed by determination of the Flack parameter. In the crystal, the mol­ecules are linked by four hydrogen bonds, one classical (N—H⋯N) and three ‘weak’ (C—H⋯O), forming layers parallel to the *ac* plane; these are in turn linked in the third dimension by Cl⋯N [3.1689 (16) Å] and Cl⋯O [3.3148 (13) Å] contacts to the heterocyclic ring.

## Related literature

For the chemotherapeutic effects of substituted-1,3,4-oxadiazole derivatives, see: Aboraia *et al.* (2006 ▶); Akhtar *et al.* (2008 ▶, 2010 ▶); Khan *et al.* (2005 ▶); Mishra *et al.* (2005 ▶); Zahid *et al.* (2009 ▶). Based on the known structures of 2,5-disubstituted-1,3,4-oxadiazo­les with diverse biological activity, we have designed and synthesized several new derivatives of 1,3,4-oxadiazo­les and evaluated their anti-HIV activity, see: Syed *et al.* (2011 ▶).
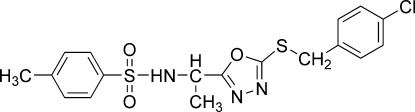


## Experimental

### 

#### Crystal data

C_18_H_18_ClN_3_O_3_S_2_
*M* *_r_* = 423.92
Orthorhombic, 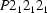

*a* = 5.5928 (3) Å
*b* = 17.5004 (7) Å
*c* = 20.1431 (7) Å
*V* = 1971.53 (15) Å^3^
*Z* = 4
Cu *K*α radiation
μ = 3.90 mm^−1^
*T* = 100 K
0.15 × 0.10 × 0.06 mm

#### Data collection

Oxford Diffraction Xcalibur Nova A diffractometer
Absorption correction: multi-scan (*CrysAlis PRO*; Oxford Diffraction, 2010 ▶) *T*_min_ = 0.785, *T*_max_ = 1.000
31188 measured reflections
3762 independent reflections
3625 reflections with *I* > 2σ(*I*)
*R*_int_ = 0.046

#### Refinement

*R*[*F* ^2^ > 2σ(*F* ^2^)] = 0.026
*wR*(*F* ^2^) = 0.069
*S* = 1.04
3762 reflections
250 parameters
H atoms treated by a mixture of independent and constrained refinement
Δρ_max_ = 0.31 e Å^−3^
Δρ_min_ = −0.28 e Å^−3^
Absolute structure: Flack (1983 ▶), 1563 Friedel pairs
Flack parameter: −0.001 (11)

### 

Data collection: *CrysAlis PRO* Oxford Diffraction (2010 ▶); cell refinement: *CrysAlis PRO*; data reduction: *CrysAlis PRO*; program(s) used to solve structure: *SHELXS97* (Sheldrick, 2008 ▶); program(s) used to refine structure: *SHELXL97* (Sheldrick, 2008 ▶); molecular graphics: *XP* (Siemens, 1994 ▶) and *RPLUTO* (CCDC, 2007 ▶); software used to prepare material for publication: *SHELXL97*.

## Supplementary Material

Crystal structure: contains datablock(s) I, global. DOI: 10.1107/S1600536811039134/bt5653sup1.cif

Structure factors: contains datablock(s) I. DOI: 10.1107/S1600536811039134/bt5653Isup2.hkl

Supplementary material file. DOI: 10.1107/S1600536811039134/bt5653Isup3.cml

Additional supplementary materials:  crystallographic information; 3D view; checkCIF report

## Figures and Tables

**Table 1:** Hydrogen-bond geometry (Å, °)

*D*—H⋯*A*	*D*—H	H⋯*A*	*D*⋯*A*	*D*—H⋯*A*
N5—H05⋯N4^i^	0.90 (3)	2.19 (3)	3.068 (2)	166 (2)
C15—H15*B*⋯O2^ii^	0.99	2.41	3.301 (2)	149
C9—H9⋯O2^iii^	0.95	2.43	3.178 (2)	136
C15—H15*B*⋯O3^iv^	0.99	2.47	3.007 (2)	113

Symmetry codes: (i) 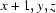; (ii) 
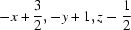
; (iii) 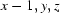; (iv) 
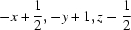
.

**Fig. 1.**
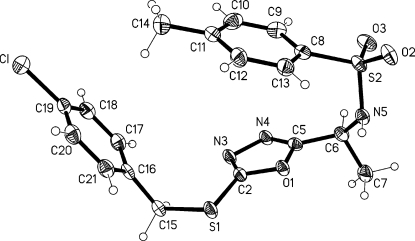
The molecule of the title compound. Ellipsoids represent 50% probability levels.

**Fig. 2.**
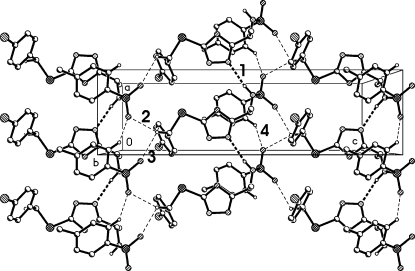
Molecular packing of the title compound viewed parallel to the b axis in the region y≈ 1/2.. Thick dashed lines represent classical and thin dashed lines "weak" hydrogen bonds. The numbering corresponds to the order in the H bond Table. H atoms not involved in H bonds are omitted.

**Fig. 3.**
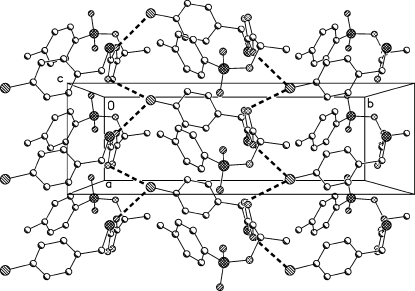
Molecular packing of the title compound viewed parallel to the c axis in the region z≈ 1/8. A l l H atoms are omitted. The thick dashed lines represent Cl···O and Cl···N contacts.

## supplementary crystallographic information

### Comment

Substituted-1,3,4-oxadiazole derivatives are of significant interest because of their chemotherapeutic effects such as anti-proliferative (Zahid *et al.*, 2009), anti-tumour and anti-viral (Akhtar *et al.*, 2008), anti-microbial (Mishra *et al.*, 2005), urease inhibition (Akhtar *et al.*, 2010), tyrosinase inhibition (Khan *et al.*, 2005), and anti-mitotic (Aboraia *et al.*, 2006) activities. Based on the known structures of 2,5-disubstituted-1,3,4-oxadiazoles with diverse biological activities, we have designed and synthesized several new derivatives of 1,3,4-oxadiazoles and evaluated their anti-HIV activity (Syed *et al.*, 2011). In this paper, we   report the crystal structure of one of these compounds.

The molecule of the enantiomerically pure title compound is shown in Fig. 1. Bond lengths and angles may be regarded as normal. The molecule has considerable potential for flexibility; the shape actually adopted is that of a short cylinder or disc, albeit distorted, in which the rings form part of the circumference. The smallest dimension of the molecular "box" is calculated by the program RPLUTO (CCDC, 2007) as 6.8 Å, which is close to the calculated distance between the *para* H atoms of a phenyl group (including van der Waals' radii). All three rings are planar within r.m.s. deviations of < 0.01 Å; interplanar angles are 29.51 (7)° and 63.43 (7)° from the five-membered ring to the aromatic systems C8–13 and C16–21 respectively, and 34.80 (6)° between these two latter rings. To close the circumference of the cylinder, the methyl hydrogen H14C approaches the centroid of the ring C16–21 at a distance of 3.08 Å.

The molecular packing is determined by four hydrogen bonds, one classical and three "weak" (including a three-centre system based on H15B), which link the molecules to form layers parallel to the *ac* plane (Fig. 2). It can be seen that the Cl atoms project out of this plane (the angle between the bond C19—Cl and the plane is 72°) and the Cl atoms thereby form short contacts Cl···N3 3.1689 (16) Å, operator -*x*, *y* - 1/2, -*z* + 1/2, and Cl···O1 3.3148 (13) Å, operator -*x* + 1, *y* - 1/2, -*z* + 1/2, to the oxadiazole ring, thus linking the layers (Fig. 3). The approximately linear angle C19—Cl···N3 166.31 (8)° is consistent with the description of Cl···N3 as a halogen bond.

### Experimental

The title compound was prepared according to a reported procedure (Syed *et al.*, 2011) and recrystallized from acetone/water.

### Refinement

The hydrogen at N5 was refined freely. Methyl H atoms were identified in difference syntheses, idealized and refined using rigid groups allowed to rotate but not tip, with C—H 0.98 Å, H—C—H 109.5°. Other H atoms were introduced at the calculated positions and refined using a riding model, with aromatic C—H 0.95, methylene C—H 0.99, methine C—H 1.00 Å. The *U*_iso_(H) values were set equal to *mU*_eq_(C) of the parent carbons, with *m* = 1.5 for methyls and 1.2 for all other H.

The absolute configuration (*S* at C6) was established by the Flack parameter of -0.001 (11).

### Crystal data

**Table:**

C_18_H_18_ClN_3_O_3_S_2_	*D*_x_ = 1.428 Mg m^−^^3^
*M**_r_* = 423.92	Cu *K*α radiation, λ = 1.54184 Å
Orthorhombic, *P*2_1_2_1_2_1_	Cell parameters from 23563 reflections
*a* = 5.5928 (3) Å	θ = 3.3–75.8°
*b* = 17.5004 (7) Å	µ = 3.90 mm^−^^1^
*c* = 20.1431 (7) Å	*T* = 100 K
*V* = 1971.53 (15) Å^3^	Tablet, colourless
*Z* = 4	0.15 × 0.10 × 0.06 mm
*F*(000) = 880	

### Data collection

**Table:**

Oxford Diffraction Xcalibur Nova A diffractometer	3762 independent reflections
Radiation source: Nova (Cu) X-ray Source	3625 reflections with *I* > 2σ(*I*)
mirror	*R*_int_ = 0.046
Detector resolution: 10.3543 pixels mm^-1^	θ_max_ = 70.2°, θ_min_ = 3.4°
ω–scan	*h* = −6→6
Absorption correction: multi-scan (*CrysAlis PRO*; Oxford Diffraction, 2010)	*k* = −21→20
*T*_min_ = 0.785, *T*_max_ = 1.000	*l* = −24→24
31188 measured reflections	

### Refinement

**Table:**

Refinement on *F*^2^	Secondary atom site location: difference Fourier map
Least-squares matrix: full	Hydrogen site location: inferred from neighbouring sites
*R*[*F*^2^ > 2σ(*F*^2^)] = 0.026	H atoms treated by a mixture of independent and constrained refinement
*wR*(*F*^2^) = 0.069	*w* = 1/[σ^2^(*F*_o_^2^) + (0.0449*P*)^2^ + 0.4932*P*] where *P* = (*F*_o_^2^ + 2*F*_c_^2^)/3
*S* = 1.04	(Δ/σ)_max_ = 0.001
3762 reflections	Δρ_max_ = 0.31 e Å^−^^3^
250 parameters	Δρ_min_ = −0.28 e Å^−^^3^
0 restraints	Absolute structure: Flack (1983), 1563 Friedel pairs
Primary atom site location: structure-invariant direct methods	Flack parameter: −0.001 (11)

### Special details

**Table:**

Geometry. All e.s.d.'s (except the e.s.d. in the dihedral angle between two l.s. planes) are estimated using the full covariance matrix. The cell e.s.d.'s are taken into account individually in the estimation of e.s.d.'s in distances, angles and torsion angles; correlations between e.s.d.'s in cell parameters are only used when they are defined by crystal symmetry. An approximate (isotropic) treatment of cell e.s.d.'s is used for estimating e.s.d.'s involving l.s. planes.Non-bonded contacts:3.1689 (0.0016) Cl - N3_$5 3.3148 (0.0013) Cl - O1_$6 3.7719 (0.0007) Cl - S1_$6166.31 (0.08) C19 - Cl - N3_$5 102.19 (0.11) Cl - N3_$5 - C2_$5 126.37 (0.07) C19 - Cl - O1_$6 109.03 (0.09) Cl - O1_$6 - C2_$6 114.16 (0.06) C19 - Cl - S1_$6 83.66 (0.06) Cl - S1_$6 - C2_$6Operators for generating equivalent atoms: $5 - *x*, *y* - 1/2, -*z* + 1/2 $6 - *x* + 1, *y* - 1/2, -*z* + 1/2===============================================================Least-squares planes (*x*,*y*,*z* in crystal coordinates) and deviations from them (* indicates atom used to define plane)2.6855 (0.0038) *x* - 4.6429 (0.0122) *y* + 16.8416 (0.0087) *z* = 2.0416 (0.0053)* -0.0119 (0.0012) C16 * 0.0104 (0.0013) C17 * 0.0005 (0.0013) C18 * -0.0099 (0.0013) C19 * 0.0082 (0.0013) C20 * 0.0027 (0.0013) C21Rms deviation of fitted atoms = 0.0084- 1.1391 (0.0042) *x* + 17.0266 (0.0034) *y* - 2.2001 (0.0174) *z* = 7.9763 (0.0084)Angle to previous plane (with approximate e.s.d.) = 63.43 (0.07)* -0.0027 (0.0009) O1 * 0.0047 (0.0010) C2 * -0.0046 (0.0010) N3 * 0.0027 (0.0010) N4 * -0.0002 (0.0010) C5Rms deviation of fitted atoms = 0.00342.6434 (0.0040) *x* - 12.9885 (0.0091) *y* + 9.5712 (0.0144) *z* = 1.4590 (0.0089)Angle to previous plane (with approximate e.s.d.) = 29.51 (0.07)* 0.0016 (0.0013) C8 * -0.0004 (0.0013) C9 * 0.0015 (0.0014) C10 * -0.0039 (0.0014) C11 * 0.0051 (0.0015) C12 * -0.0040 (0.0014) C13Rms deviation of fitted atoms = 0.00322.6855 (0.0038) *x* - 4.6429 (0.0122) *y* + 16.8416 (0.0087) *z* = 2.0416 (0.0053)Angle to previous plane (with approximate e.s.d.) = 34.80 (0.08)* -0.0119 (0.0012) C16 * 0.0104 (0.0013) C17 * 0.0005 (0.0013) C18 * -0.0099 (0.0013) C19 * 0.0082 (0.0013) C20 * 0.0027 (0.0013) C21Rms deviation of fitted atoms = 0.0084
Refinement. Refinement of *F*^2^ against ALL reflections. The weighted *R*-factor *wR* and goodness of fit *S* are based on *F*^2^, conventional *R*-factors *R* are based on *F*, with *F* set to zero for negative *F*^2^. The threshold expression of *F*^2^ > σ(*F*^2^) is used only for calculating *R*-factors(gt) *etc*. and is not relevant to the choice of reflections for refinement. *R*-factors based on *F*^2^ are statistically about twice as large as those based on *F*, and *R*- factors based on ALL data will be even larger.

### Fractional atomic coordinates and isotropic or equivalent isotropic displacement parameters (Å^2^)

**Table:**

	*x*	*y*	*z*	*U*_iso_*/*U*_eq_	
Cl	0.05592 (10)	0.18862 (3)	0.15994 (2)	0.03349 (12)	
S1	0.52403 (8)	0.54474 (3)	0.263650 (19)	0.02387 (11)	
S2	0.77730 (8)	0.45463 (3)	0.555955 (19)	0.02286 (11)	
O1	0.5636 (2)	0.55679 (7)	0.39301 (5)	0.0211 (3)	
O2	1.0271 (3)	0.44179 (8)	0.56672 (6)	0.0292 (3)	
O3	0.6148 (3)	0.45159 (8)	0.61070 (6)	0.0303 (3)	
C2	0.4061 (3)	0.54027 (10)	0.34332 (8)	0.0193 (3)	
N3	0.1914 (3)	0.52799 (9)	0.36370 (7)	0.0235 (3)	
N4	0.2027 (3)	0.53824 (9)	0.43387 (7)	0.0229 (3)	
C5	0.4199 (3)	0.55439 (9)	0.44771 (8)	0.0202 (3)	
N5	0.7624 (3)	0.54058 (9)	0.52475 (7)	0.0221 (3)	
H05	0.879 (5)	0.5469 (14)	0.4944 (12)	0.034 (6)*	
C6	0.5302 (3)	0.57714 (10)	0.51284 (8)	0.0225 (4)	
H6	0.4186	0.5626	0.5495	0.027*	
C7	0.5667 (4)	0.66361 (11)	0.51427 (9)	0.0311 (4)	
H7A	0.6287	0.6788	0.5578	0.047*	
H7B	0.4137	0.6892	0.5062	0.047*	
H7C	0.6813	0.6783	0.4797	0.047*	
C8	0.6782 (3)	0.38998 (10)	0.49450 (9)	0.0236 (4)	
C9	0.4622 (4)	0.35176 (11)	0.50209 (10)	0.0289 (4)	
H9	0.3651	0.3602	0.5401	0.035*	
C10	0.3915 (4)	0.30098 (11)	0.45291 (11)	0.0332 (5)	
H10	0.2447	0.2743	0.4577	0.040*	
C11	0.5301 (4)	0.28842 (11)	0.39702 (10)	0.0317 (4)	
C12	0.7460 (4)	0.32732 (12)	0.39112 (9)	0.0334 (5)	
H12	0.8441	0.3185	0.3534	0.040*	
C13	0.8207 (4)	0.37862 (11)	0.43917 (9)	0.0278 (4)	
H13	0.9671	0.4056	0.4343	0.033*	
C14	0.4505 (5)	0.23359 (13)	0.34315 (11)	0.0460 (6)	
H14A	0.3121	0.2043	0.3588	0.069*	
H14B	0.5816	0.1985	0.3325	0.069*	
H14C	0.4062	0.2625	0.3033	0.069*	
C15	0.2572 (4)	0.52048 (10)	0.21572 (8)	0.0243 (4)	
H15A	0.1158	0.5422	0.2384	0.029*	
H15B	0.2691	0.5450	0.1716	0.029*	
C16	0.2174 (3)	0.43593 (10)	0.20602 (8)	0.0217 (4)	
C17	0.0137 (3)	0.40140 (11)	0.23032 (9)	0.0267 (4)	
H17	−0.0945	0.4301	0.2568	0.032*	
C18	−0.0354 (4)	0.32510 (11)	0.21652 (9)	0.0273 (4)	
H18	−0.1774	0.3020	0.2328	0.033*	
C19	0.1239 (4)	0.28349 (11)	0.17903 (9)	0.0249 (4)	
C20	0.3328 (4)	0.31573 (11)	0.15569 (9)	0.0284 (4)	
H20	0.4433	0.2861	0.1308	0.034*	
C21	0.3790 (3)	0.39220 (11)	0.16906 (9)	0.0264 (4)	
H21	0.5218	0.4150	0.1529	0.032*	

### Atomic displacement parameters (Å^2^)

**Table:**

	*U*^11^	*U*^22^	*U*^33^	*U*^12^	*U*^13^	*U*^23^
Cl	0.0429 (3)	0.0264 (2)	0.0311 (2)	−0.00061 (19)	−0.0014 (2)	−0.00327 (18)
S1	0.0258 (2)	0.0332 (2)	0.01268 (18)	−0.00294 (18)	0.00137 (15)	−0.00194 (16)
S2	0.0256 (2)	0.0304 (2)	0.01258 (18)	0.00513 (18)	0.00069 (15)	0.00086 (15)
O1	0.0218 (6)	0.0281 (6)	0.0133 (5)	−0.0008 (5)	0.0012 (5)	−0.0009 (5)
O2	0.0297 (7)	0.0400 (7)	0.0179 (6)	0.0082 (6)	−0.0051 (5)	−0.0023 (5)
O3	0.0356 (8)	0.0373 (7)	0.0180 (6)	0.0076 (6)	0.0067 (5)	0.0046 (5)
C2	0.0219 (9)	0.0211 (8)	0.0150 (7)	0.0014 (6)	−0.0008 (6)	0.0002 (6)
N3	0.0288 (9)	0.0297 (8)	0.0121 (7)	−0.0004 (6)	0.0004 (6)	0.0000 (5)
N4	0.0251 (8)	0.0292 (8)	0.0144 (6)	−0.0015 (7)	0.0012 (6)	0.0013 (6)
C5	0.0242 (9)	0.0207 (8)	0.0155 (8)	0.0024 (7)	0.0033 (7)	0.0013 (6)
N5	0.0215 (8)	0.0292 (8)	0.0154 (7)	−0.0007 (6)	0.0006 (6)	−0.0004 (6)
C6	0.0243 (10)	0.0268 (9)	0.0162 (8)	0.0011 (7)	0.0025 (7)	0.0002 (6)
C7	0.0442 (12)	0.0287 (10)	0.0205 (9)	0.0013 (9)	−0.0028 (8)	−0.0057 (7)
C8	0.0266 (10)	0.0238 (9)	0.0202 (8)	0.0032 (7)	−0.0008 (7)	0.0038 (7)
C9	0.0259 (10)	0.0289 (9)	0.0320 (10)	0.0040 (8)	0.0047 (8)	0.0044 (7)
C10	0.0264 (11)	0.0277 (9)	0.0454 (12)	−0.0036 (8)	−0.0050 (8)	0.0063 (8)
C11	0.0428 (12)	0.0263 (9)	0.0259 (9)	−0.0046 (9)	−0.0113 (9)	0.0055 (7)
C12	0.0459 (13)	0.0366 (11)	0.0175 (8)	−0.0087 (9)	0.0011 (8)	−0.0015 (7)
C13	0.0319 (11)	0.0328 (10)	0.0187 (8)	−0.0066 (8)	0.0035 (7)	0.0016 (7)
C14	0.0648 (16)	0.0385 (11)	0.0349 (11)	−0.0163 (11)	−0.0161 (12)	0.0023 (9)
C15	0.0284 (10)	0.0299 (9)	0.0146 (8)	0.0006 (7)	−0.0036 (7)	0.0011 (7)
C16	0.0238 (9)	0.0300 (9)	0.0112 (7)	0.0026 (7)	−0.0036 (6)	0.0007 (6)
C17	0.0270 (10)	0.0331 (9)	0.0199 (8)	0.0032 (8)	0.0011 (7)	−0.0022 (7)
C18	0.0261 (10)	0.0347 (10)	0.0211 (8)	0.0001 (8)	0.0011 (7)	0.0012 (7)
C19	0.0314 (10)	0.0258 (9)	0.0173 (8)	0.0007 (7)	−0.0056 (7)	−0.0004 (7)
C20	0.0314 (10)	0.0335 (10)	0.0202 (8)	0.0041 (8)	0.0018 (7)	−0.0041 (7)
C21	0.0252 (10)	0.0346 (10)	0.0194 (8)	−0.0008 (7)	0.0028 (7)	−0.0003 (7)

### Geometric parameters (Å, °)

**Table:**

Cl—C19	1.7462 (19)	C16—C21	1.399 (3)
S1—C2	1.7369 (17)	C17—C18	1.391 (3)
S1—C15	1.8272 (19)	C18—C19	1.376 (3)
S2—O3	1.4300 (13)	C19—C20	1.380 (3)
S2—O2	1.4314 (15)	C20—C21	1.389 (3)
S2—N5	1.6323 (15)	C6—H6	1.0000
S2—C8	1.7662 (19)	C7—H7A	0.9800
O1—C2	1.364 (2)	C7—H7B	0.9800
O1—C5	1.365 (2)	C7—H7C	0.9800
C2—N3	1.287 (2)	C9—H9	0.9500
N3—N4	1.4262 (19)	C10—H10	0.9500
N4—C5	1.278 (2)	C12—H12	0.9500
C5—C6	1.504 (2)	C13—H13	0.9500
N5—C6	1.468 (2)	C14—H14A	0.9800
C6—C7	1.527 (3)	C14—H14B	0.9800
C8—C13	1.384 (3)	C14—H14C	0.9800
C8—C9	1.389 (3)	C15—H15A	0.9900
C9—C10	1.388 (3)	C15—H15B	0.9900
C10—C11	1.384 (3)	C17—H17	0.9500
C11—C12	1.391 (3)	C18—H18	0.9500
C11—C14	1.515 (3)	C20—H20	0.9500
C12—C13	1.385 (3)	C21—H21	0.9500
C15—C16	1.509 (2)	N5—H05	0.90 (3)
C16—C17	1.380 (3)		
			
C2—S1—C15	99.64 (8)	C20—C21—C16	120.64 (18)
O3—S2—O2	119.84 (8)	C6—N5—H05	118.5 (16)
O3—S2—N5	107.38 (8)	S2—N5—H05	109.7 (16)
O2—S2—N5	104.64 (8)	N5—C6—H6	108.7
O3—S2—C8	108.50 (9)	C5—C6—H6	108.7
O2—S2—C8	108.17 (8)	C7—C6—H6	108.7
N5—S2—C8	107.72 (8)	C6—C7—H7A	109.5
C2—O1—C5	101.85 (13)	C6—C7—H7B	109.5
N3—C2—O1	113.82 (14)	H7A—C7—H7B	109.5
N3—C2—S1	131.04 (13)	C6—C7—H7C	109.5
O1—C2—S1	115.02 (12)	H7A—C7—H7C	109.5
C2—N3—N4	104.70 (14)	H7B—C7—H7C	109.5
C5—N4—N3	106.62 (14)	C10—C9—H9	120.7
N4—C5—O1	113.00 (15)	C8—C9—H9	120.7
N4—C5—C6	129.72 (15)	C11—C10—H10	119.3
O1—C5—C6	117.03 (16)	C9—C10—H10	119.3
C6—N5—S2	120.65 (12)	C13—C12—H12	119.4
N5—C6—C5	112.98 (14)	C11—C12—H12	119.4
N5—C6—C7	108.09 (16)	C8—C13—H13	120.6
C5—C6—C7	109.49 (15)	C12—C13—H13	120.6
C13—C8—C9	121.33 (18)	C11—C14—H14A	109.5
C13—C8—S2	118.41 (15)	C11—C14—H14B	109.5
C9—C8—S2	120.27 (14)	H14A—C14—H14B	109.5
C10—C9—C8	118.51 (18)	C11—C14—H14C	109.5
C11—C10—C9	121.49 (19)	H14A—C14—H14C	109.5
C10—C11—C12	118.54 (18)	H14B—C14—H14C	109.5
C10—C11—C14	121.2 (2)	C16—C15—H15A	108.6
C12—C11—C14	120.2 (2)	S1—C15—H15A	108.6
C13—C12—C11	121.28 (19)	C16—C15—H15B	108.6
C8—C13—C12	118.84 (19)	S1—C15—H15B	108.6
C16—C15—S1	114.63 (13)	H15A—C15—H15B	107.6
C17—C16—C21	118.88 (17)	C16—C17—H17	119.6
C17—C16—C15	120.35 (17)	C18—C17—H17	119.6
C21—C16—C15	120.66 (17)	C19—C18—H18	120.3
C16—C17—C18	120.82 (17)	C17—C18—H18	120.3
C19—C18—C17	119.33 (18)	C19—C20—H20	120.5
C18—C19—C20	121.24 (18)	C21—C20—H20	120.5
C18—C19—Cl	118.89 (15)	C20—C21—H21	119.7
C20—C19—Cl	119.87 (15)	C16—C21—H21	119.7
C19—C20—C21	119.05 (17)		
			
C5—O1—C2—N3	−0.76 (19)	N5—S2—C8—C9	−110.51 (15)
C5—O1—C2—S1	175.70 (11)	C13—C8—C9—C10	0.5 (3)
C15—S1—C2—N3	−3.60 (19)	S2—C8—C9—C10	−179.52 (15)
C15—S1—C2—O1	−179.31 (13)	C8—C9—C10—C11	−0.5 (3)
O1—C2—N3—N4	0.94 (19)	C9—C10—C11—C12	0.8 (3)
S1—C2—N3—N4	−174.82 (14)	C9—C10—C11—C14	−179.29 (19)
C2—N3—N4—C5	−0.73 (19)	C10—C11—C12—C13	−1.1 (3)
N3—N4—C5—O1	0.3 (2)	C14—C11—C12—C13	178.9 (2)
N3—N4—C5—C6	174.36 (16)	C9—C8—C13—C12	−0.8 (3)
C2—O1—C5—N4	0.24 (18)	S2—C8—C13—C12	179.18 (16)
C2—O1—C5—C6	−174.64 (14)	C11—C12—C13—C8	1.1 (3)
O3—S2—N5—C6	−45.79 (14)	C2—S1—C15—C16	−86.77 (14)
O2—S2—N5—C6	−174.15 (12)	S1—C15—C16—C17	118.83 (16)
C8—S2—N5—C6	70.90 (14)	S1—C15—C16—C21	−64.89 (19)
S2—N5—C6—C5	−83.75 (17)	C21—C16—C17—C18	−2.2 (3)
S2—N5—C6—C7	154.93 (12)	C15—C16—C17—C18	174.13 (16)
N4—C5—C6—N5	138.58 (19)	C16—C17—C18—C19	1.1 (3)
O1—C5—C6—N5	−47.6 (2)	C17—C18—C19—C20	0.9 (3)
N4—C5—C6—C7	−100.9 (2)	C17—C18—C19—Cl	−178.08 (14)
O1—C5—C6—C7	73.0 (2)	C18—C19—C20—C21	−1.6 (3)
O3—S2—C8—C13	−174.53 (14)	Cl—C19—C20—C21	177.35 (14)
O2—S2—C8—C13	−43.07 (17)	C19—C20—C21—C16	0.4 (3)
N5—S2—C8—C13	69.52 (17)	C17—C16—C21—C20	1.5 (3)
O3—S2—C8—C9	5.45 (17)	C15—C16—C21—C20	−174.86 (17)
O2—S2—C8—C9	136.91 (15)		

### Hydrogen-bond geometry (Å, °)

**Table:**

*D*—H···*A*	*D*—H	H···*A*	*D*···*A*	*D*—H···*A*
N5—H05···N4^i^	0.90 (3)	2.19 (3)	3.068 (2)	166 (2)
C15—H15B···O2^ii^	0.99	2.41	3.301 (2)	149.
C9—H9···O2^iii^	0.95	2.43	3.178 (2)	136.
C15—H15B···O3^iv^	0.99	2.47	3.007 (2)	113.

Symmetry codes: (i) *x*+1, *y*, *z*; (ii) −*x*+3/2, −*y*+1, *z*−1/2; (iii) *x*−1, *y*, *z*; (iv) −*x*+1/2, −*y*+1, *z*−1/2.
